# A Strain Transfer Model for Detection of Pitting Corrosion and Loading Force of Steel Rebar with Distributed Fiber Optic Sensor

**DOI:** 10.3390/s23198142

**Published:** 2023-09-28

**Authors:** Jialiang Hu, Fujian Tang, Tianjiao Li, Gang Li, Hong-Nan Li

**Affiliations:** 1 School of Civil Engineering, Dalian University of Technology, Dalian 116024, China; 2 Deep Underground Engineering Research Center, Dalian University of Technology, Dalian 116024, China

**Keywords:** distributed fiber optic sensor, strain transfer, pitting corrosion, structural health monitoring

## Abstract

Steel rebar corrosion is one of the predominant factors influencing the durability of marine and offshore reinforced concrete structures, resulting in economic loss and the potential threat to human safety. Distributed fiber optic sensors (DFOSs) have gradually become an effective method for structural health monitoring over the past two decades. In this work, a strain transfer model is developed between a steel rebar and a DFOS, considering pitting-corrosion-induced strain variation in the steel rebar. The Gaussian function is first adopted to describe the strain distribution near the corrosion pit of the steel rebar and then is substituted into the governing equation of the strain transfer model, and the strain distribution in the DFOS is analytically obtained. Tensile tests are also conducted on steel rebars with artificially simulated corrosion pits, which are used to validate the developed model. The results show that the Gaussian function can be used to describe the strain variation near a corrosion pit with a depth less than 50% of the steel rebar diameter, and the strain distribution in the DFOS analytically determined based on the developed strain transfer model agrees well with the tensile test results. The corrosion pit depth and loading force in the steel rebars estimated based on the proposed model agree well with the actual values, and therefore, the developed strain transfer model is effective in detecting pitting corrosion and loading force in steel rebars.

## 1. Introduction

Due to easy accessibility and low cost of raw materials, reinforced concrete (RC) is one of the paramount artificial building materials in the world. It has low cost, good integrity and can be used to manufacture structural elements with any shape. However, during its entire service life, RC structural deterioration is inevitable, especially for those exposed to aggressive environments. For instance, reinforcement steel corrosion is one of the major causes of RC structural degradation in marine environments or in cold districts with frequent application of deicing salts [1,2]. Reinforcement steel corrosion leads to cracking of the concrete protective layer [3], degradation of the mechanical properties of steel rebars [4], and loss of bond between concrete and steel rebars, which results in reduction in the bearing capacity of structural members [5,6] and even collapse of RC structures [7]. To ensure the safety and reliability of RC structures during the whole service life, it is necessary to develop sensors for real-time corrosion monitoring of steel rebars.

Conventional sensors to monitor steel rebar corrosion in RC structures are either electrochemical-based methods (half-cell potential, linear polarization resistance, etc.) or physical-based techniques (acoustic emission, ultrasonic waves, etc.) [8,9]. Compared with electrochemically or physically based corrosion sensors, optical fiber sensors demonstrate the advantages of light weight, small size, high sensitivity, high resolution, corrosion resistance, immunity to electromagnetic interference, and multiplexity, which meet the needs of structural health monitoring [10]. Many optical fiber corrosion sensors based on different sensing principles have been proposed to monitor reinforcement steel corrosion in RC structures [11], such as fiber Bragg grating (FBG)-based corrosion sensors [12], long-period fiber grating (LPFG)-based corrosion sensors [13,14], extrinsic Fabry–Perot interferometer (EFPI)-based corrosion sensors [15], single-mode–multimode–single-mode fiber-based corrosion sensors [16], Brillouin optical time-domain analysis (BOTDA)-based sensors, and optical frequency domain reflectometry (OFDR)-based sensors [17,18,19,20]. Since reinforcement steel corrosion in RC structures is random, it initiates and propagates nonuniformly in space and over time. Therefore, distributed fiber optic sensors are more advantageous than point-based fiber optic sensors in this regard.

Many researchers have investigated distributed optical fiber sensors (DFOSs) for corrosion monitoring of steel rebars over the past two decades. According to different monitoring objects, the utilization of a DFOS to monitor steel rebar corrosion is generally classified into direct and indirect methods. A DFOS is wrapped directly around the steel rebar to measure corrosion-induced volume expansion according to the direct method [21], while it is wrapped on the surface of the concrete cover to measure corrosion-induced concrete tensile strain or cracking for the indirect method [17]. A relationship needs to be established between DFOS strain and steel rebar corrosion, which in turn is used to monitor the corrosion degree of steel rebar.

Localized corrosion or pitting corrosion is the most prevalent type of corrosion that occurs in steel rebar for RC structures in a chloride-rich environment. Compared with generalized corrosion, pitting corrosion is more dangerous. It causes stress concentration and strain variation for both RC surfaces and steel bars. There are many strain sensors to monitor strain, such as traditional strain gauges and dual-interrogation-mode radio frequency identification (RFID) strain sensors [22,23]. However, these sensors only measure the localized strain, which cannot reflect the strain distribution along the length of the entire steel bar. If a DFOS is installed along the longitudinal ridge of a steel rebar that is subjected to stress, pitting corrosion would cause strain variation in the DFOS at the location of pitting corrosion [24], and the strain variation would be closely related to the size of the pitting corrosion when the steel rebar is in the elastic stage. Therefore, it would be possible to detect pitting corrosion occurring in a steel rebar with a DFOS if the strain monitored by the DFOS is the strain in the corroded steel rebar. However, the installation of a DFOS introduces intermediate layers (coating, adhesive layer) between steel rebars and DFOSs [25], and the deformation of the intermediate layers, as well as the interfacial slip between them, influence the transfer of strain from steel rebars to DFOSs [26].

Many strain transfer models have been proposed to describe the transfer of substrate strain to optical fiber sensors in the past three decades. Based on the assumptions that all materials are linear elastic and all interfaces are perfectly bonded, Ansari et al. [27] proposed a strain transfer model from the structural host to the optical fiber core and found that the strain transfer depended on the mechanical properties of each material layer and the sticking length of the optical fiber. To account for the loss of strain in the protective coating of optical fiber, Li et al. [28] developed a strain transfer model by considering the fiber coating as an elastoplastic material. Huang et al. [29] analytically derived the strain transfer rate from axially loaded inelastic concrete due to the optical fiber sensor, which was packaged in a glass-fiber-reinforced polymer. Slips may occur at the interface between the optical fiber and fiber coating. Zhao et al. [30] proposed a simplified trilinear three-stage interfacial bond-slip model between the optical fiber and fiber coating and developed a strain transfer model for large crack monitoring. In addition, sensitivity analyses have also been conducted to explore the influences of adhesive thickness [31,32], adhesive length [32,33,34], binder stiffness [33,34,35,36], binder selection [37], different host materials [38], and different types of optical fiber sensors [39] on the strain transfer efficiency. The above-mentioned researchers discuss strain transfer under uniform strain fields. There are also some scholars who have investigated strain transfer effects under a nonuniform field. Tan et al. [40] quantitatively evaluated the strain transfer for a DFOS subjected to an arbitrary strain field. Bassil et al. [41] applied more adequate boundary conditions to the traditional strain transfer model, and the new model allowed analysis under any arbitrary strain field.

However, few studies discuss the strain transfer effect between DFOSs and steel rebars with pitting corrosion. Therefore, to monitor pitting corrosion in steel rebars based on the strain variation obtained from a DFOS, it is necessary to develop a strain transfer model between a DFOS and a steel rebar with pitting corrosion, which in turn can be used to back-calculate the size of pitting corrosion, as well as the strain level in the steel rebar.

This work aims to develop a strain transfer model between a steel rebar with pitting corrosion and a DFOS and, further, to evaluate the size of pitting corrosion and loading force in the steel rebar. First, a small groove is cut along the longitudinal ridge of a steel rebar, and then a DFOS is embedded in the groove with epoxy resin. In the meantime, one V-shaped cut with five different depths is made in the middle of the steel rebar to simulate different sizes of pitting corrosion. Second, tensile tests are conducted, during which the DFOS strain distribution is recorded under different loading forces. Third, finite element models of the steel rebar are established, and the steel rebar strain distribution at the location of the DFOS is obtained, along the length, which is compared with DFOS strain curves. Fourth, a Gaussian function is proposed to fit the strain distribution of steel rebars obtained numerically and then is substituted into the governing equation of strain transfer. The analytical strain distribution function of the DFOS is determined by solving the governing equation. Fifth, the parameters in the Gaussian function are determined by fitting the analytical strain distribution function of the DFOS with those obtained during tensile tests, and then the size of the simulated pitting corrosion and loading force in the steel rebar are estimated and compared with the actual values.

## 2. Materials and Methods

### 2.1. Specimen Praparation

**Figure 1:**
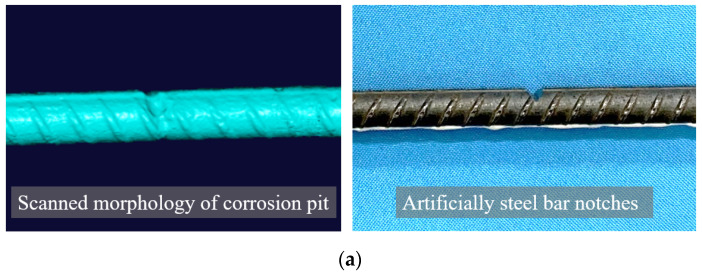

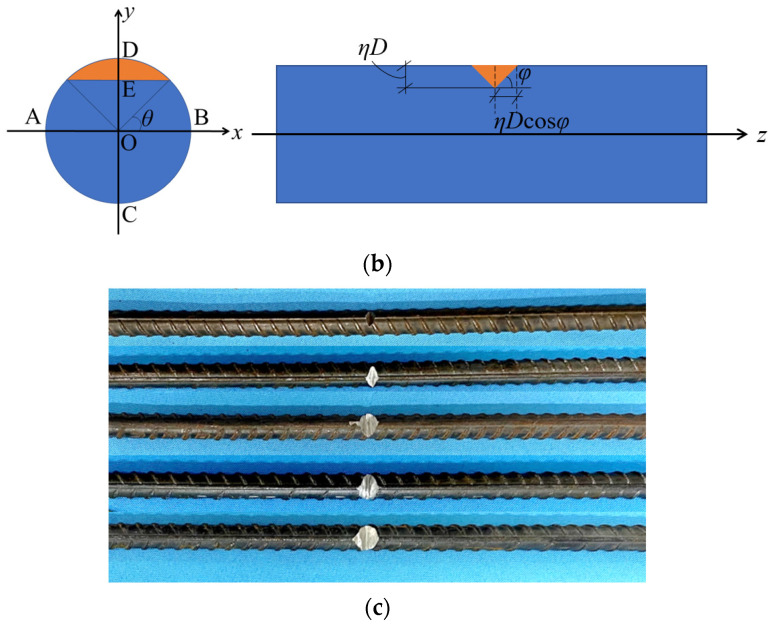
(**a**) Comparison between actual corrosion pit and artificial V-shaped cut; (**b**) dimensions of V-shaped cut in steel rebar; (**c**) steel rebars with different sizes of V-shaped cuts.

Hot-rolled ribbed steel bars (HRB 400) with a diameter of 12 mm and a length of 500 mm were used in this study. The yield strength and elastic modulus of the steel rebars were measured to be 400 MPa and 180 GPa, respectively. In order to quantitatively describe the strain transfer law between the matrix and the optical fiber at the pit position of the local corroded steel bar more accurately, one V-shaped cut was made in the middle of the steel rebars to simulate pitting corrosion, as shown in Figure 1a,b. To consider different sizes of pitting corrosion, a total of five different depths of V-shaped cuts were made, which corresponded to 10%, 20%, 30%, 40%, and 50% of the rebar diameter, respectively, as shown in Figure 1b. The residual cross-sectional area and the mass loss of steel rebars at the section of the V-shaped cut were determined and are listed in Table 1.

### 2.2. Installation of DFOS

**Figure 2:**
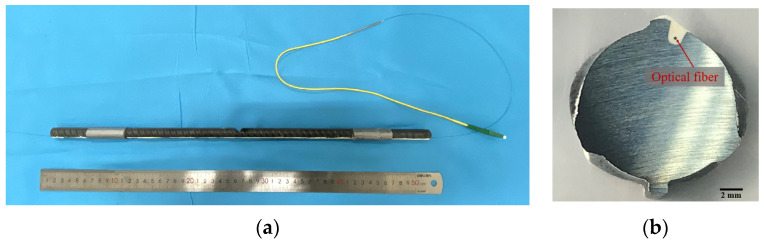
(**a**) Installation of a DFOS in a precut groove of a steel rebar; (**b**) cross-sectional view of a steel rebar instrumented with a DFOS in the groove.

Commercially available single-mode optical fiber was used as a distributed fiber optical sensor (DFOS), which consisted of cladding and a core with a diameter of 125 µm and 9 µm, respectively. The DFOS had an outer layer of polyimide coating that protected it from damage. To install the DFOS along the lengths of steel bars, a small groove was precut along the longitudinal ridge of each steel rebar. The dimensions of the precut groove were around 1.0 mm × 2.0 mm, as shown in Figure 2. Marine epoxy resin was used to seal the optical fiber, as shown in Figure 2a. The cross-section of the sealed steel rebar is shown in Figure 2b. The properties of the DFOS and epoxy are listed in Table 2.

### 2.3. Tensile Tests

**Figure 3:**
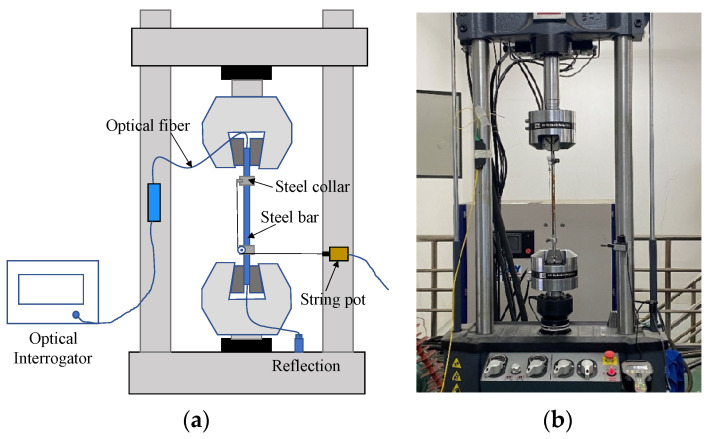
(**a**) Schematic illustration of tensile test; (**b**) tensile test in the lab.

Tensile tests were performed on steel rebars using an MTS machine (MTS Systems Corporation, MN, USA) with displacement control, as schematically shown in Figure 3a. Displacement-controlled loading was applied at a loading rate of 70 µm/minute. During the tensile test, the strain of the DFOS was measured using optical frequency domain reflectometry (OFDR), Luna ODiSI-B (Luna Innovations, VA, USA). The specifications of the MTS machine and OFDR are listed in Table 3. A specially designed apparatus was also used to measure the deformation of steel rebars around the V-shaped cut zone, as shown in Figure 3a. Two steel collars were fabricated and fixed at the two ends of the measured section (see Figure 3b). The elongation of the section was recorded using a string potentiometer (Milang Technology, Shenzhen, China) with a resolution of 10 µm.

## 3. Numerical Simulation

**Figure 4:**
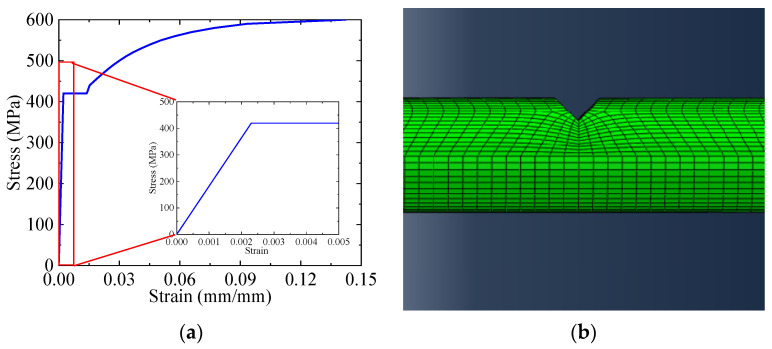
(**a**) Constitutive law of steel rebars used in the FE model; (**b**) FE model of a steel rebar with a 20% cut depth.

To obtain the strain distribution of steel rebars at the location of the embedded DFOSs, finite element (FE) models were also constructed using Abaqus software. The constitutive law of steel rebars used in the FE analysis is shown in Figure 4a, and Figure 4b shows the FE model of a steel rebar near V-shaped cut with a depth of 50% of the diameter. The eight-node linear brick element was used, and a total of ~130,000 elements were used for a steel rebar.

## 4. Results

### Strain Distribution of the DFOS

**Figure 5:**
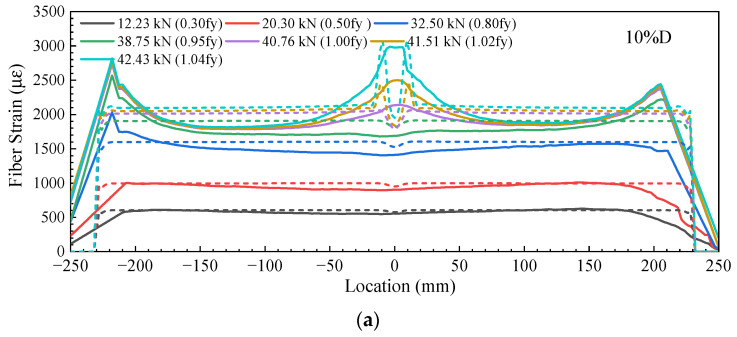

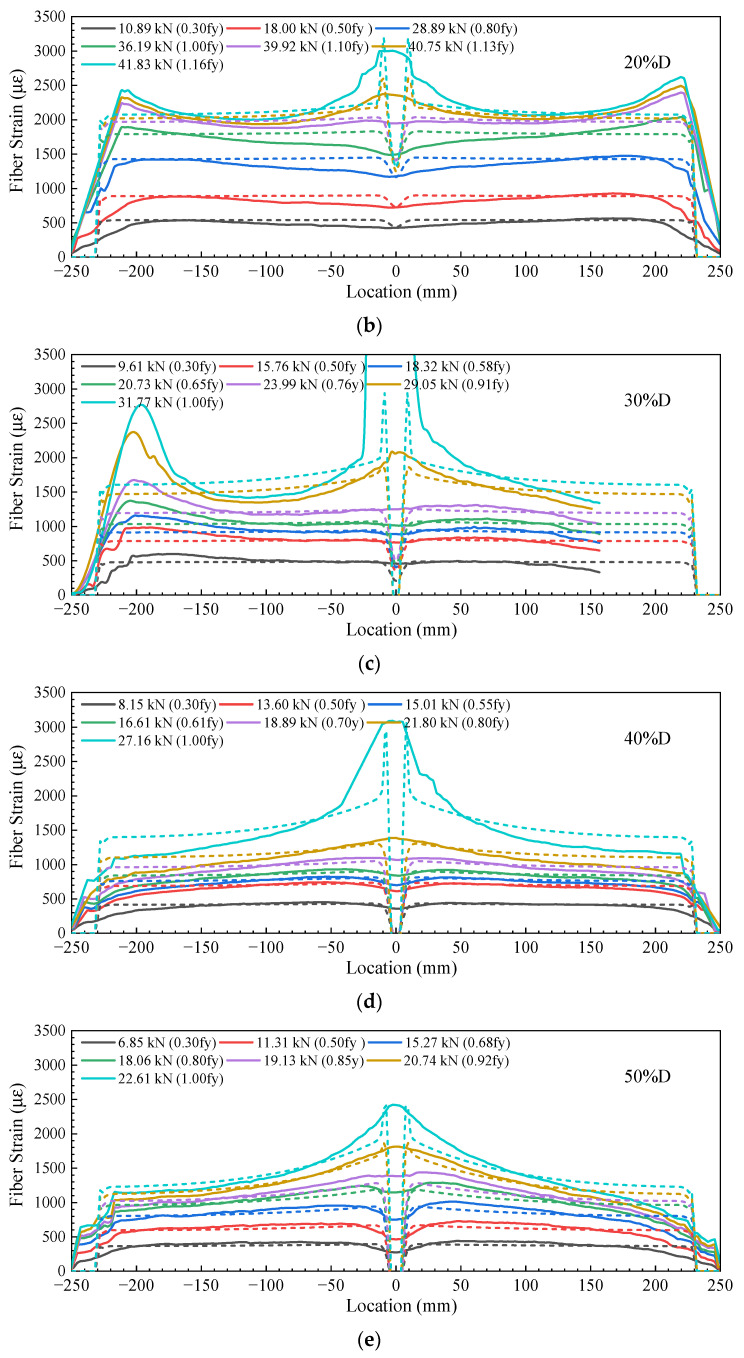
OFDR strain distribution for steel bars with pit depths of (**a**) 10% of D, (**b**) 20% of D, (**c**) 30% of D, (**d**) 40% of D, and (**e**) 50% of D at different loading levels. (The solid lines represent OFDR-measured strain data; the dash lines represent FE-simulated strains).

Figure 5 shows the distribution of DFOS strain along the length of steel rebars under different loading levels, and the fiber strain was not constantly distributed along the length. For the steel rebar with a cut depth of 10% of the diameter, as shown in Figure 5a, the DFOS strains were nearly constant along the length and had an average value of ~617 µε when the loading force was 12.23 kN. It increased to 1000 µε as the loading force increased up to 20.30 kN. A small dip started to appear at the location of the V-shaped cut as the loading force increased up to 32.50 kN and continued to be present as the loading force reached 38.75 kN. The appearance of the small dip is attributed to the deviation in the loading center from the geometric center of the residual cross-section of the steel rebar, and therefore, the tensile stress in the DFOS, which was far from the geometric center, was compensated for with the bending compression. However, as the loading force increased to 40.76 kN, the dip turned to become a strain peak. At this level of loading, the measured DFOS strain was over 2000 µε, indicating yielding of the steel rebar at the V-shaped cut location. Once the yield of the steel rebar was initiated, plastic deformation occurred at the simulated pitting corrosion section and led to redistribution of tensile stress and, consequently, tensile stress concentration at this section. As the loading force continued to increase, the strains at the V-shaped cut section increased rapidly and extended to the adjoining regions, while the strains increased slightly at other locations far from the V-shaped cut location. For instance, the DFOS peak strain increased from 2100 µε to 3000 µε at the V-shaped cut section, while the strains far from the section remained stabilized at around 1800 µε as the loading force increased from 40.76 kN to 42.43 kN.

For comparison, the FE-simulated strains at the location of the DFOS are also shown by the dashed lines in Figure 5a. It can be seen that, while the loading force was at low levels, i.e., smaller than 20.30 kN, the DFOS strains were consistent with the FE results in general. However, when the loading force reached 32.50 kN, the DFOS strain values were significantly different from the FE results near the V-shaped cut section. This difference increased with an increase in the loading force because local yielding at the section generated a sharp increase in rebar deformation. The local increase in deformation was transferred to DFOS elongation through the epoxy resin. Consequently, the DFOS deformation was extended to the surrounding region since the interfaces between the epoxy and steel rebar, as well as those between the epoxy resin and the DFOS, were not perfectly bonded. It should be pointed out that the higher strain values at the two ends were attributed to the combined stress from both tensile loading and compression from the clamp of the MTS machine. The sections away from the machine clamp and the V-shaped cut location were lower than the actual values.

As the depth of the V-shaped cut increased to 20% and 30% of the diameter of the steel rebars, as shown in Figure 5b,c, a similar trend of DFOS strain distribution was observed as that of the steel rebar with the 10% V-shaped depth. However, as the V-shaped depth increased up to 40% and 50% of the diameter of the steel rebar, the strain dip at the V-shaped location appeared at very low loading levels, as shown in Figure 5d,e. Moreover, the strain dip became a strain peak at low loading levels (i.e., 21.80 kN for the 40% depth cut and 19.13 kN for the 50% depth cut). In addition, the extended width of the strain peak was larger than that of steel rebars with 10% and 20% cut depths. This different behavior is attributed to the effect of eccentricity: the stress state was complicated, and stress concentration became dominant in steel rebars. Moreover, the stress in the DFOS started to redistribute along the length due to incomplete shear stress transfer between the steel rebar and the DFOS in the presence of the fiber coating and epoxy resin layer.

## 5. Strain Transfer Model

### 5.1. Strain Distribution and Strain Transfer of Steel Rebars with Pitting Corrosion

It can be observed from the previous section that there are significant differences between the OFDR-measured strain data and the FE-simulated strain distribution, especially near the V-shaped cut section. The FE-simulated strains are considered to be the actual strain of the steel rebar at the location of the DFOS, while the measured strain is the deformation of the DFOS, which is transferred from the steel rebar through the epoxy resin and the fiber coating layer. Before deriving the equation for strain transfer, the strain distribution in the steel rebars at regions near the V-shaped cut is modeled with a Gaussian function, which is mathematically expressed as follows:

(1) $$\varepsilon_{h}\left(x\right)=a+\frac{b}{c\sqrt{\frac{\pi }{2}}}e^{\frac{-2x^{2}}{c^{2}}}$$

where *a*, *b*, and *c* are parameters that can be obtained via curve fitting; $\varepsilon_{h}$ is the strain of the steel rebar host; and x is the point location in the steel rebar.

**Figure 6:**
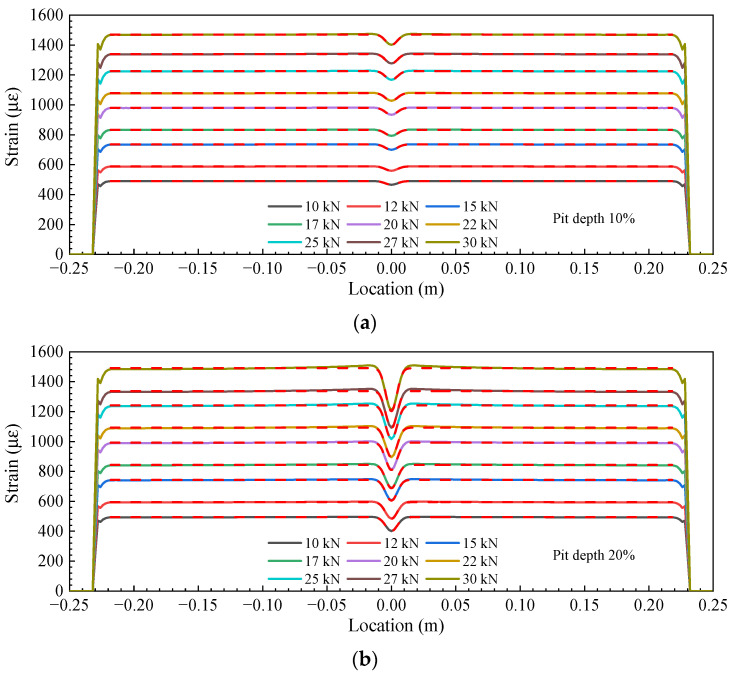

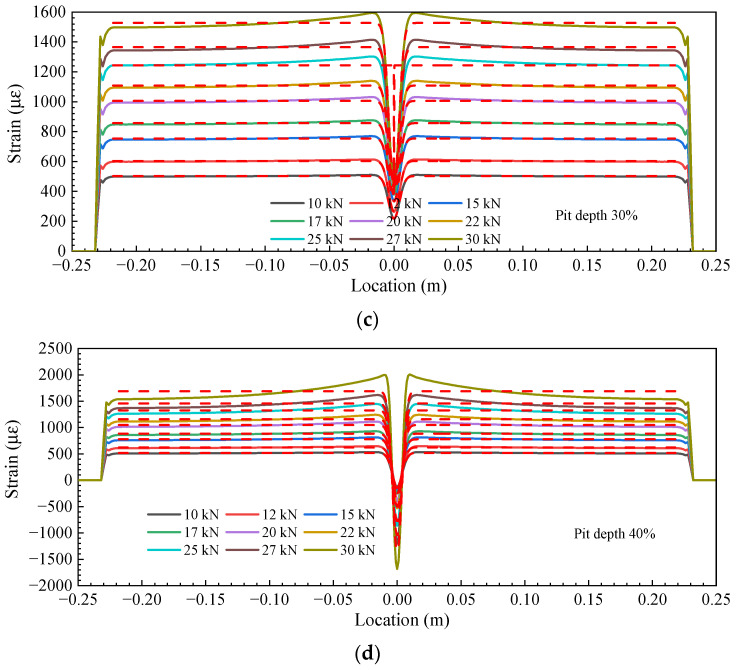
The FE-simulated strain distribution (solid lines) and the fitted curves (dash lines) based on Equation (1) for steel rebars with pit depths of (**a**) 10% of the D, (**b**) 20% of the D, (**c**) 30% of the D, and (**d**) 40% of the D.

Figure 6 compares the FE-simulated strain curve in the steel rebars with cut depths ranging from 10% to 40% of the diameter and the fitted curves from Equation (1). It should be pointed out that the fitting error for the steel bar with a cut depth of 50% of the D was large, and therefore, the Gaussian function was not suitable for this case. The solid lines are the FE results, and the dashed lines are the fitting results. It can be seen that, when the depth of the V-shaped cut was 10% and 20% of the D, the fitted curves agreed well with the FE results. As the cut depth increased to 30% and 40%, some slight differences were observed.

Based on the parameters of fitted curves in Figure 6, the mathematical expressions of both the pit depth and the loading force in the steel rebar as a function of Gaussian parameters *a* and *b* are given as follows:

(2) $$\begin{array}{l} D=14.16-7.67\times 10^{-2}a+1.77\times 10^{-4}a^{2}-2.01\times 10^{-7}a^{3}+1.09\times 10^{-10}a^{4}-2.3\times 10^{-14}a^{5}\\ -1.24b-1.91\times 10^{-1}b^{2}-1.56\times 10^{-2}b^{3}-6.08\times 10^{-4}b^{4}-8.81\times 10^{-6}b^{5},\;\;R^{2}=0.980 \end{array}$$

(3) $$\begin{array}{l} F=-8.01\times 10^{-3}+2.12\times 10^{-2}a-2.91\times 10^{-6}a^{2}+4.56\times 10^{-9}a^{3}-3.25\times 10^{-12}a^{4}+8.37\times 10^{-16}a^{5}\\ +1.44\times 10^{-1}b-1.61\times 10^{-2}b^{2}+1.91\times 10^{-3}b^{3}+9.20\times 10^{-5}b^{4}+1.59\times 10^{-6}b^{5},\;\;R^{2}=0.999 \end{array}$$

The *R*^2^ of the two fitting surfaces were both greater than 0.980, indicating that the fitting accuracy could be improved by considering both parameters *a* and *b*, and satisfactory pit depth and loading force could be estimated with high accuracy without considering parameter *c*. Therefore, in the following sections, parameter *c* is directly assigned as 0.00813, which is the average value of parameter *c* in Figure 6.

Equations (2) and (3) provide functions for predicting pit depth and load by Gaussian parameters *a*, *b*, and *c*. Then, for physical tensile tests, a new fitted equation derived based on the strain transfer theory should be used to fit the measured fiber strain distribution to get the Gaussian parameters. The process of obtaining the fitted equation is as follows:

As the DFOS was embedded in the host material, all the intermediate materials were assumed to be linearly elastic, and the governing equation of strain transfer from the host material to the DFOS can be expressed as follows [40,41]:

(4) $$\varepsilon_{f}{}^{\prime \prime }\left(x\right)-k^{2}\varepsilon_{f}\left(x\right)+k^{2}\varepsilon_{h}\left(x\right)=0$$

where $\varepsilon_{f}$ and $\varepsilon_{h}$ are, respectively, the strains of the DFOS and host material (steel rebar in this study). The interfaces between them were assumed to be perfectly bonded in this study. Then, *k* is a constant that is dependent on the dimensions and material properties of the DFOS, which is written as follows [40,41]:

(5) $$k^{2}=\frac{2}{E_{f}r_{f}^{2}\left[\frac{\ln\left(r_{c}/r_{f}\right)}{G_{c}}+\frac{\ln\left(r_{e}/r_{c}\right)}{G_{e}}\right]}$$

where $r_{f}$, $r_{c}$, and $r_{e}$ are the radius of the DFOS, fiber coating, and epoxy resin, respectively. $E_{f}$ is the elastic modulus of the DFOS. $G_{c}$ and $G_{e}$ are the shear modulus of the fiber coating and epoxy resin, respectively.

When substituting Equation (1) into Equation (2), the governing equation is as follows:

(6) $$\varepsilon_{f}{}^{\prime \prime }\left(x\right)-k^{2}\varepsilon_{f}\left(x\right)+k^{2}\left(a+\frac{b}{c\sqrt{\frac{\pi }{2}}}e^{\frac{-2x^{2}}{c^{2}}}\right)=0$$

The solution of differential Equation (4) consists of a complementary solution and a particular solution. The complementary solution can be obtained by solving the following:

(7) $$\frac{d^{2}\varepsilon_{f}\left(x\right)}{dx^{2}}-k^{2}\varepsilon_{f}\left(x\right)=0$$

Assuming a solution of $\varepsilon_{f}\left(x\right)=e^{\lambda x}$, it can be substituted into Equation (7):

(8) $$\lambda^{2}e^{\lambda x}-k^{2}e^{\lambda x}=0$$

Since $e^{\lambda x}\neq 0$, then

(9) $$\lambda^{2}-k^{2}=0$$

Thus,

(10) λ=±k

Therefore, the general solution is as follows:

(11) $$\varepsilon_{f}\left(x\right)=\alpha_{1}e^{-kx}+\alpha_{2}e^{kx}$$

where $\alpha_{1}$ and $\alpha_{2}$ are constants.

The particular solution of Equation (6) can be obtained via variation of parameters. List the basis solutions in $\varepsilon_{\mathrm{fc}}\left(x\right)$:

(12) $$\varepsilon_{\mathrm{fb}1}\left(x\right)=e^{-kx}\;\mathrm{and}\;\varepsilon_{\mathrm{fb}1}\left(x\right)=e^{kx}$$

The Wronskian of $\varepsilon_{\mathrm{fb}1}\left(x\right)$ and $\varepsilon_{\mathrm{fb}1}\left(x\right)$ is as follows:

(13) $$W\left(x\right)=\left|\begin{array}{c} e^{-kx}\\ \frac{d}{dx}\left(e^{-kx}\right) \end{array}\right.\left.\begin{array}{c} e^{kx}\\ \frac{d}{dx}\left(e^{kx}\right) \end{array}\right|=\left|\begin{array}{c} e^{-kx}\\ -ke^{-kx} \end{array}\right.\left.\begin{array}{c} e^{kx}\\ ke^{kx} \end{array}\right|=2k$$

Let

(14) $$f\left(x\right)=-k^{2}\left(a+\frac{b}{c\sqrt{\frac{\pi }{2}}}e^{\frac{-2x^{2}}{c^{2}}}\right)$$

and

(15) $$v_{1}\left(x\right)=-\int \frac{f\left(x\right)\varepsilon_{\mathrm{fb}2}\left(x\right)}{W\left(x\right)}dx$$

(16) $$v_{2}\left(x\right)=\int \frac{f\left(x\right)\varepsilon_{\mathrm{fb}1}\left(x\right)}{W\left(x\right)}dx$$

The particular solution can be given with the following:

(17) $$\varepsilon_{\mathrm{fp}}\left(x\right)=v_{1}\left(x\right)\varepsilon_{\mathrm{fb}1}\left(x\right)+v_{2}\left(x\right)\varepsilon_{\mathrm{fb}2}\left(x\right)$$

where

(18) $$v_{1}\left(x\right)=-\int -\frac{1}{2}ke^{kx}\left(a+\frac{b}{c\sqrt{\frac{\pi }{2}}}e^{\frac{-2x^{2}}{c^{2}}}\right)dx=\frac{k}{2}\left(\frac{ae^{kx}}{k}-\frac{1}{2}be^{\frac{c^{2}k^{2}}{8}}\mathrm{erf}\left(\frac{c^{2}k-4x}{2\sqrt{2}c}\right)\right)$$

(19) $$v_{2}\left(x\right)=\int -\frac{1}{2}ke^{-kx}\left(a+\frac{b}{c\sqrt{\frac{\pi }{2}}}e^{\frac{-2x^{2}}{c^{2}}}\right)dx=-\frac{k}{2}\left(-\frac{ae^{-kx}}{k}-\frac{1}{2}bk\cdot e^{\left(\frac{c^{2}k^{2}}{8}+kx\right)}\mathrm{erf}\left(\frac{c^{2}k+4x}{2\sqrt{2}c}\right)\right)$$

Thus, the particular solution is as follows:

(20) $$\varepsilon_{\mathrm{fp}}\left(x\right)=\frac{k}{2}e^{-kx}\left(\frac{ae^{kx}}{k}-\frac{1}{2}be^{\frac{c^{2}k^{2}}{8}}\mathrm{erf}\left(\frac{c^{2}k-4x}{2\sqrt{2}c}\right)\right)-\frac{k}{2}e^{kx}\left(-\frac{ae^{-kx}}{k}+\frac{1}{2}be^{\frac{c^{2}k^{2}}{8}}\mathrm{erf}\left(\frac{c^{2}k+4x}{2\sqrt{2}c}\right)\right)$$

which is further simplified as

(21) $$\varepsilon_{\mathrm{fp}}\left(x\right)=a-\frac{1}{4}bk\cdot e^{\left(\frac{c^{2}k^{2}}{8}-kx\right)}\mathrm{erf}\left(\frac{c^{2}k-4x}{2\sqrt{2}c}\right)-\frac{1}{4}bk\cdot e^{\left(\frac{c^{2}k^{2}}{8}+kx\right)}\mathrm{erf}\left(\frac{c^{2}k+4x}{2\sqrt{2}c}\right)$$

Therefore, the solution of Equation (4) is expressed as follows:

(22) $$\varepsilon_{f}\left(x\right)=a+\alpha_{1}\cdot e^{kx}+\alpha_{2}\cdot e^{-kx}-\frac{1}{4}bk\cdot e^{\left(\frac{c^{2}k^{2}}{8}-kx\right)}\mathrm{erf}\left(\frac{c^{2}k-4x}{2\sqrt{2}c}\right)-\frac{1}{4}bk\cdot e^{\left(\frac{c^{2}k^{2}}{8}+kx\right)}\mathrm{erf}\left(\frac{c^{2}k+4x}{2\sqrt{2}c}\right)$$

Parameters $\alpha_{1}$ and $\alpha_{2}$ can be determined using the boundary conditions of $\varepsilon_{f}\left(-0.25\right)=\varepsilon_{f}\left(0.25\right)=0$, and then the strain distribution in the DFOS is as follows:

(23) $$\begin{array}{l} \varepsilon_{f}\left(x\right)=a+\frac{-a+\frac{1}{4}bk\cdot e^{\frac{c^{2}k^{2}}{8}}\left(e^{\frac{k}{4}}-e^{-\frac{k}{4}}\right)}{e^{\frac{k}{4}}+e^{-\frac{k}{4}}}\cdot (e^{kx}+e^{-kx})-\frac{1}{4}bk\cdot e^{\left(\frac{c^{2}k^{2}}{8}-kx\right)}\mathrm{erf}\left(\frac{c^{2}k-4x}{2\sqrt{2}c}\right)\\ -\frac{1}{4}bk\cdot e^{\left(\frac{c^{2}k^{2}}{8}+kx\right)}\mathrm{erf}\left(\frac{c^{2}k+4x}{2\sqrt{2}c}\right) \end{array}$$

### 5.2. Model Validation

**Figure 7:**
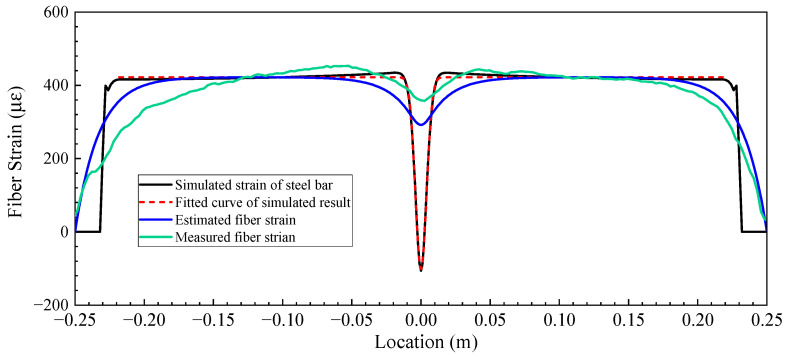
The strain distribution of a steel rebar and the DFOS strain based on the model with a pit depth of 4.8 mm and a loading force of 8.15 kN.

Equation (23) will be used to fit the measured fiber strain distribution. The steel rebar with a cut depth of 4.8 mm and a loading force of 8.15 kN was used as an example to validate the proposed strain transfer model considering pitting corrosion in steel rebar. According to the fitted Gaussian function, a=422.79, b=−5.46, and c=0.0085. The constant k used in this validation equaled 58.65, which was calculated based on Equation (4) according to the properties of the DFOS and epoxy, as shown in Table 2. The results are shown in Figure 7, and the FE-simulated and Gaussian-function-fitted strain curves of the steel rebar are also shown for comparison. It can be observed that the strain curve of the DFOS analytically obtained based on the proposed strain transfer model was similar to that of the DFOS embedded in the steel rebar. Therefore, the proposed strain transfer model was able to describe the fiber strain transfer for steel rebar with pitting corrosion. However, there remained an apparent difference between the analytical curve and the measured curve, which was mainly caused by the following reasons: First, the proposed Gaussian function was unable to fully represent the strain distribution of steel rebars with pit cuts, especially when the depth of the pit was greater than 40% of the diameter. Second, the strain transfer model assumed linear elastic materials for the fiber coating and epoxy resin, as well as a perfect bond between them (the value of parameter k), which was not the case in reality [27,28,29,30]. Third, there were some differences between the FE-simulated strain curve of the steel rebar and the actual strain curve of the steel rebar during tensile tests, as the pit bottom in the FE model was sharper and there was a certain weakening in the real steel rebar. Finally, the steel rebar was not perfectly in tension during the tests, and there might be some eccentric tension.

## 6. Estimation of Pit Depth and Loading Force

The purpose of the proposed strain transfer model is to estimate the pit depth and the loading force in the steel rebar based on the strain curve of a DFOS embedded in the steel rebar. It should be noted that the analytical strain distribution obtained from the proposed strain transfer model in Equation (23) was a function of Gaussian function parameters *a*, *b*, and *c*, which were dependent on the pit depth and loading force in the steel rebar. Therefore, relationships needed to be established between the Gaussian parameters and the pit depth, as well as the loading force in the steel rebar, which in turn could be used to estimate the pit depth and loading force in the steel rebar based on the readings of the DFOS. Based on the fitted parameters in Figure 6, the mathematical expressions of both the pit depth and the loading force in the steel rebar as a function of Gaussian parameters *a* and *b* are given as Equations (2) and (3).

**Figure 8:**
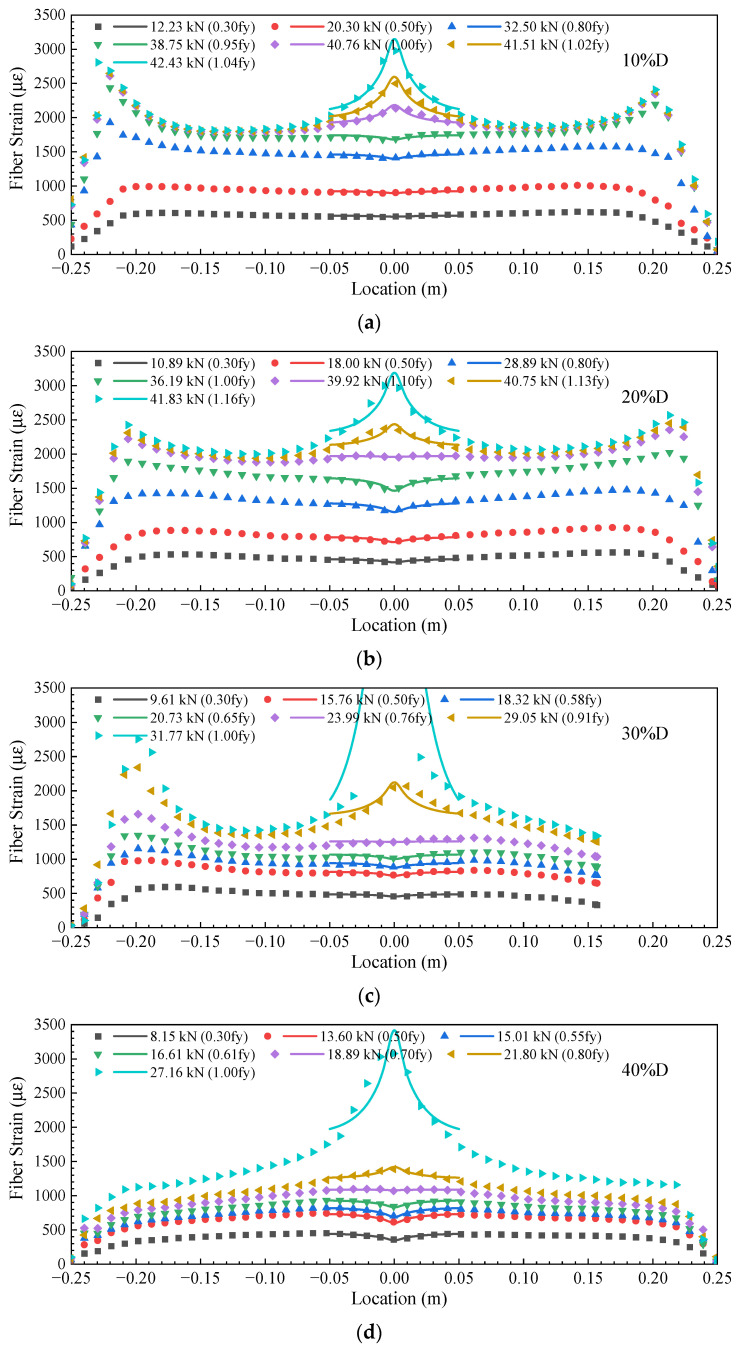

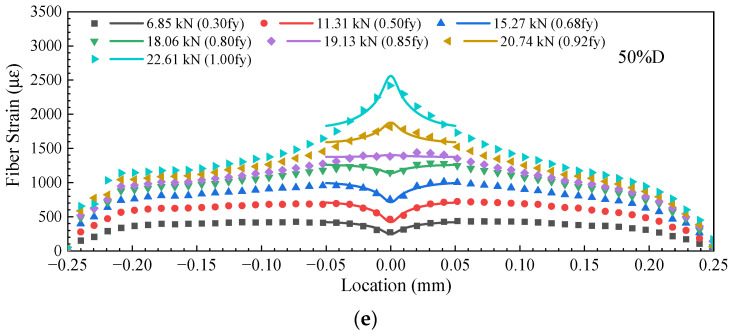
The DFOS strain distribution and the fitted curves based on Equation (19) of steel rebars with pit depths of (**a**) 10% of the D, (**b**) 20% of the D, (**c**) 30% of the D, (**d**) 40%of the D, and (**e**) 50% of the D. The dots represent the DFOS strain, and the solid lines represent the fitted curves for the location between [−0.05, 0.05].

To verify the accuracy of Equations (2) and (3), the analytical strain distribution function obtained from the developed strain transfer model in Equation (23) was used to fit all DFOS strain readings for steel rebars with cut depths ranging from 10% to 40% of the D, and Figure 8 shows the results. The dots represent the DFOS strain, and the solid lines represent the fitted curves for a segment between [−0.05, 0.05]. It can be seen that the fitted strain curves agreed well with the measured DFOS strain curves.

**Figure 9:**
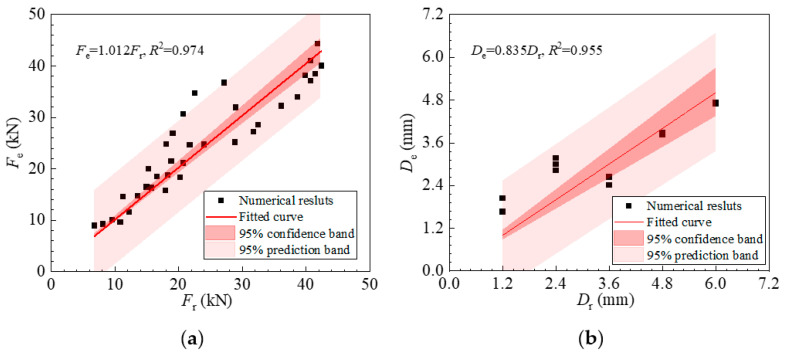
Comparison of (**a**) loading force and (**b**) pit depth estimated based on the proposed strain transfer model and the real values.

The fitted Gaussian function parameters were substituted into Equations (22) and (23) to obtain the estimated pit depth and loading force in the steel rebars. Figure 9 compares the estimated loading force and pit depth based on the proposed strain transfer model and the corresponding real values, as well as the 95% confidence band and 95% prediction band. The horizontal axis represents the true value, and the vertical axis represents the estimated value based on the DFOS results using the proposed strain transfer model. The 95% confidence band is used to estimate the range of likely values for population parameters, while the 95% prediction band is wider and is used to estimate the range of likely values for individual predictions in predictive modeling. It can be seen that the estimated loading force and pit depth agreed with the actual values despite some errors, the source of which was discussed in Section 5.2. Therefore, the developed strain transfer mode could be used to estimate both the depth of the pit corrosion and the loading force in the steel rebars by applying a Gaussian function to describe the strain distribution near the corrosion pit sections.

## 7. Application of the Strain Transfer Model on the Estimation of Actual Corrosion Pit

**Figure 10:**
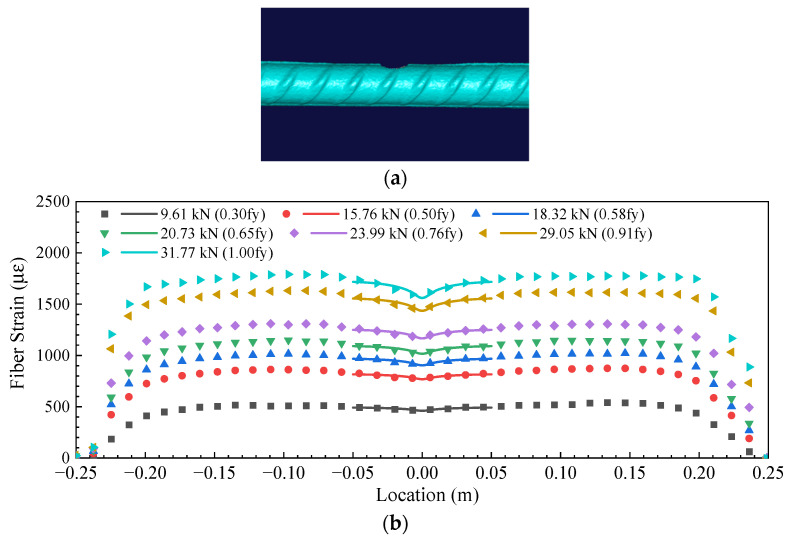
(**a**) A scanned figure and (**b**) the DFOS strain distribution and the fitted curves based on Equation (23) of the pitting corrosion induced by accelerated corrosion of hydrochloric acid.

Hydrochloric acid was used to accelerate the corrosion of steel bars to create a pit. The scanned figure of the final pitting corrosion is shown in Figure 10a. Then, a uniaxial tensile test was carried out on the steel rebar. The method proposed in this paper is used to predict the corrosion pit: (i) Use OFDR to measure the strain distribution in the fiber; (ii) Apply Equation (23) to fit the strain distribution curve and get the fitted parameters a, b and c, as shown in Figure 10. (iii) Calculate the estimated value based on Equations (2) and (3). (iv) Revised the estimated value by the euqtions listed in Figure 9. The final estimated value and measured value of pit depth and force are listed in Table 4. The relative errors of the estimated force and pit depth are less than 2.82% and 12.4%, respectively. 

## 8. Conclusions

In this work, a strain transfer model between a DFOS and a steel bar with a corrosion pit was developed. A Gaussian function was substituted into the governing equation of the strain transfer to describe the pit-induced strain variation. The proposed strain transfer model was validated via tensile tests of steel rebars with different depths of V-shaped cuts. The main conclusions of this research are summarized as follows:

- A strain transfer model was developed between a corroded steel rebar and a DFOS considering pitting-corrosion-induced strain variation in the steel rebar, and the strain distribution in the DFOS was analytically determined by solving the governing equation of the strain transfer model.
- A Gaussian function was proposed to fit the strain distribution near the corrosion pit in the steel rebar, and the fitting parameters were dependent on the loading force and the pit depth.
- The strain distribution of the DFOS analytically obtained based on the proposed strain transfer model agreed well with the actual strain distribution of the DFOSs embedded in the steel rebars during tensile tests.
- The pit depth and loading force in the steel rebars were estimated based on the proposed strain transfer model, and the estimated values agreed well with the actual pit depth and loading force.
- The source of errors between the estimated and the actual pit depth or loading force were mainly from (i) the simplified strain distribution near the corrosion pit of steel rebars with the Gaussian function; (ii) the assumed linear elasticity of the intermediate materials between the steel rebars and the DFOS, as well as a perfect bond between them; and (iii) the difference between the FE-simulated strain and the actual strain in the steel rebars during tensile tests.

Although the strain transfer model was effective in detecting the corrosion depth and loading force of steel bars, there are some limitations: (1) the proposed strain transfer model was only applicable to a single corrosion pit; (2) the steel bars were in the elastic tension stage; (3) the pit depth should be less than or equal to 40% of the steel bar diameter; and (4) the corrosion pits were artificially V-shaped, which is different from the actual shape of corrosion pits in engineering practice. In the future, a more generalized strain transfer model should be developed that is able to detect multiple corrosion pits when a steel bar is in both elastic and plastic stages and the corrosion depth is greater than 40% of the steel bar diameter. Experimental work should be conducted on reinforced concrete structural members (i.e., beams, columns, etc.) subjected to different times of corrosion.

## Figures and Tables

**Table 1:** Residual cross-sectional areas of steel bars with different sizes of V-shaped cuts.

Depth of V-Shaped Cut (D%) (mm)	Residual Cross-Sectional Area (mm^2^)	Mass Loss (%)
1.2 (10%)	107	5.2
2.4 (20%)	97	14.2
3.6 (30%)	85	25.2
4.8 (40%)	71	37.4
6.0 (50%)	57	50.0

**Table 2:** Geometrical and mechanical properties of the DFOS and epoxy.

Material	Radius (μm)	Shear Modulus (GPa)	Poisson’s Ratio
Fiber core and cladding	62.5	28.6	0.26
Fiber coating	95	7.0 × 10^−4^	0.42
Epoxy	1000	2.1	0.35

**Table 3:** Specifications of MTS and OFDR.

Machine Name	Parameter	Specification
MTS	Model number	MTS 370.10
	Dynamic load capacity	100 kN
	Static load capacity	120 kN
	Test height	140 mm~1283 mm
LUNA	Model number	ODiSI-B10
	Maximum sensing length	10 m
	Wavelength accuracy	1.5 pm
	Acquisition rate	100 Hz
	Strain range	±10,000 µStrain
	Strain repeatability	±5 µStrain
	Gage length	2.61 mm

**Table 4:** Comparison between the estimated value and measured value of pit depth and force of the steel rebar with a pit induced by accelerated corrosion of hydrochloric acid.

Force			Pit Depth		
Measured Value (kN)	Estimated Value (kN)	Relative Error	Measured Value (mm)	Estimated Value (mm)	Relative Error
9.61	9.99	3.99%	3.21	2.81	12.40%
15.76	16.09	2.10%	3.21	2.85	11.21%
18.32	18.96	3.49%	3.21	3.02	5.99%
20.73	21.29	2.72%	3.21	3.07	4.34%
23.99	24.28	1.20%	3.21	3.14	2.33%
29.05	30.05	3.44%	3.21	2.86	10.89%

## Data Availability

Some or all of the data that support the findings of this study are available from the corresponding author upon reasonable request (tensile test results and DFOS strain data).
